# Rapidly-growing mycobacterial infection: a recognized cause of early-onset prosthetic joint infection

**DOI:** 10.1186/s12879-017-2926-3

**Published:** 2017-12-28

**Authors:** Anupop Jitmuang, Varah Yuenyongviwat, Keerati Charoencholvanich, Methee Chayakulkeeree

**Affiliations:** 1 Division of Infectious Diseases and Tropical Medicine, Department of Medicine, Faculty of Medicine Siriraj Hospital, Mahidol University, 2 Wanglang Road, Bangkoknoi, Bangkok, 10700 Thailand; 2 Department of Orthopedic Surgery and Physical Medicine, Faculty of Medicine, Prince of Songkla University, Songkla, Thailand; 3 Department of Orthopedic Surgery, Faculty of Medicine Siriraj Hospital, Mahidol University, Bangkok, Thailand

**Keywords:** Hip arthroplasty, Knee arthroplasty, Mycobacteria, Prosthetic joint infection

## Abstract

**Background:**

Prosthetic joint infection (PJI) is a major complication of total hip and total knee arthroplasty (THA, TKA). Although mycobacteria are rarely the causative pathogens, it is important to recognize and treat them differently from non-mycobacterial infections. This study aimed to compare the clinical characteristics, associated factors and long-term outcomes of mycobacterial and non-mycobacterial PJI.

**Methods:**

We conducted a retrospective case-control study of patients aged ≥18 years who were diagnosed with PJI of the hip or knee at Siriraj Hospital from January 2000 to December 2012. Patient characteristics, clinical data, treatments and outcomes were evaluated.

**Results:**

A total of 178 patients were included, among whom 162 had non-mycobacterial PJI and 16 had mycobacterial PJI. Rapidly growing mycobacteria (RGM) (11) and *M. tuberculosis* (MTB) (5) were the causative pathogens of mycobacterial PJI. PJI duration and time until onset were significantly different between mycobacterial and non-mycobacterial PJI. Infection within 90 days of arthroplasty was significantly associated with RGM infection (OR 21.86; 95% CI 4.25–112.30; *p* < .001). Implant removal was associated with improved favorable outcomes at 6 months (OR 5.96; 95% CI 1.88–18.88; *p* < .01) and 12 months (OR 3.96; 95% CI 1.15–13.71; *p* = .03) after the infection.

**Conclusions:**

RGM were the major pathogens of early onset PJI after THA and TKA. Both a high clinical index of suspicion and mycobacterial cultures are recommended when medically managing PJI with negative cultures or non-response to antibiotics. Removal of infected implants was associated with favorable outcomes.

**Electronic supplementary material:**

The online version of this article (10.1186/s12879-017-2926-3) contains supplementary material, which is available to authorized users.

## Background

Total hip arthroplasty (THA) and total knee arthroplasty (TKA) can significantly improve a patient’s quality of life and are cost-effective orthopedic surgeries [1–3]. Prosthetic joint infection (PJI) is a major complication following THA and TKA, with an incidence of 0.57% to 1.7% [4, 5]. Infections that occur within 90 days of arthroplasty are classified as early onset PJI, whereas infections that occur several months to several years after arthroplasty are considered delayed or late onset PJI [6]. Risk factors associated with PJI include comorbidities such as obesity, diabetes mellitus, neoplasm, immunosuppression, postoperative complications, revision arthroplasty and a history of PJI [6]. Gram-positive cocci, especially staphylococci (60%), gram-negative bacteria (6%), anaerobes (4%), fungi (1%) and polymicrobial (20%) account for PJI pathogens, and 7% of cases are culture-negative [6, 7]. Mycobacteria, such as rapidly growing mycobacteria (RGM) and *Mycobacterium tuberculosis* (MTB), are rare causes of PJI [8]. When PJI is suspected, synovial fluid or periprosthetic tissue samples are mainly obtained for bacterial culture before giving empirical antibiotics directed towards common pathogens. Routine bacterial cultures are not ideal techniques for mycobacterial isolation, and can potentially lead to false negatives and delayed treatment. It is important to differentiate between mycobacterial and non-mycobacterial joint infections early, as the treatment of mycobacterial PJI is significantly different from that of non-mycobacterial infections. Comparisons between the clinical and treatment outcomes of mycobacterial and non-mycobacterial PJI have never been studied. This study aimed to compare the clinical manifestations, infection etiologies, treatments and long-term outcomes of mycobacterial PJI with those of non-mycobacterial PJI, and to identify the factors predictive of the outcome of mycobacterial PJI at 6 and 12 months after diagnosis.

## Methods

### Study design, data source, and population

We conducted a retrospective case-control study on patients aged ≥18 years who were diagnosed with PJI following THA or TKA at Siriraj Hospital, a large tertiary referral center in Bangkok, Thailand. All information regarding the case and control groups was derived from a retrospective chart review of patients that received treatment for PJI from January 2000 to December 2012. Based on criteria published by the Infectious Diseases Society of America (IDSA) in 2013, subjects were enrolled if there was clinical or histopathological evidence of PJI as defined elsewhere [9]. General demographic and relevant clinical data, including specific antimicrobial regimens and treatment outcomes, were recorded to compare clinical characteristics between mycobacterial PJI and PJI caused by other pathogens, and to study the factors associated with mycobacterial PJI including factors predicting favorable clinical outcomes. Inflammatory markers such as erythrocyte sedimentation rate (ESR) and C-reactive protein (CRP) and peripheral and synovial white blood cell (WBC) counts at the time of the initial clinical presentation were also recorded if available. Synovial fluid aspirations including any intra-operative synovial fluid or periprosthetic tissue samplings were performed by an orthopedic surgeon and sent for culture at the Department of Microbiology at our institution. If mycobacteria were suspected, request would be sent to the laboratory for additional mycobacterial culture using the same specimens for routine bacterial culture. Subjects who were diagnosed on December 2012 were followed the outcomes until the end of December 2013. The study was approved by Siriraj Institutional Review Board (Certificate of Approval Number SI323/2010), Faculty of Medicine Siriraj Hospital, Mahidol University, Thailand. According to a retrospective design of the study, informed consent was waived and the authors had permission to access the data of patients in this study.

### Definitions

Mycobacterial PJI was defined as the presence of any of the traditional clinical criteria [9], plus an infection confirmed microbiologically using a Lowenstein-Jensen agar or mycobacterial growth indicator tube (MGIT) (Becton Dickinson, Franklin Lakes, NJ, USA) system, biochemical testing, or the sequencing of the bacterial 16S rRNA gene, including the INNO-LiPA Mycobacteria v2 assay (Innogenetics, Ghent, Belgium). Non-mycobacterial PJI was defined as synovial fluid or periprosthetic tissue cultures positive for other pathogens, as well as clinical or pathologic criteria consistent with PJI. The onset of PJI after prosthesis implantation was classified as early-onset (within 90 days), delayed-onset (> 90–730 days), or late-onset (> 730 days) [9]. Empirical antimicrobial therapy was based on the typical dosing regimen for a susceptible pathogen, and was refined based on drug susceptibility patterns. Treatment outcomes were classified as favorable or unfavorable. Favorable outcomes were defined as follows: 1) *remission*: clinical cure and normal inflammatory markers, namely ESR or CRP; 2) *stable*: clinical cure with ESR or CRP having not returned to normal, or having been incompletely evaluated. Unfavorable outcomes were defined as follows: 1) *failure*: no clinical or microbiologic response to treatment or a severe adverse reaction that prevents the patient from continuing treatment; 2) *relapse*: persistent or recurrent infection by the same organism during antibiotic therapy or after the completion of antibiotics; 3) *re-infection*: recurrent infection by a new organism during antibiotic therapy or after completion of antibiotics; and 4) *death*: death related to PJI without other causes of infection. Treatment outcomes were evaluated at 6- and 12-month clinical follow-up appointments after hospital discharge. Subjects who were transferred out of the hospital or were lost to follow-up were excluded.

### Statistical analysis

All statistical analyses were performed using SPSS Statistics version 18.0 (SPSS, Inc., Chicago, IL, USA). Values are expressed as mean ± standard deviation or median (min, max) for continuous variables and as a frequency and percentage for categorical variables. Between-group comparisons of continuous variables were performed using a Student’s two-tailed t-test. A Mann-Whitney U test was employed for non-parametric testing. A Chi-square test and Fisher’s exact test were used to analyze differences between categorical variables. Variables found to be associated with mycobacterial PJI and with a favorable outcome by univariate analysis (*p* < .2) were included in a multivariate logistic regression model. All statistical tests were two-sided, with a *p*-value < .05 considered to be statistically significant. Outcome data were censored if a patient was lost to follow-up or transferred to another hospital.

## Results

During 12-year period of study, there were a total of 6344 TKA and 903 THA operations. A total of 178 subjects were diagnosed with PJI (2.5%), of whom 16 had mycobacterial PJI (5 from MTB, 11 from RGM) and 111 had culture-proven non-mycobacterial PJI. In overall patients with PJIs, TKA was associated with PJI in 62.2% of cases (95% CI 54.8%–69.5%), whereas THA was the site of infection in 38.7% (95% CI 30.5%–45.2%). Primary degenerative joint disease was the major pre-existing joint disorder among all subjects. The diagnosis of mycobacterial PJI was determined using periprosthetic tissue cultures (100%). RGM were a leading etiology of mycobacterial PJI. Thus, the study compared clinical characteristics between RGM PJI and culture-proven non-mycobacterial PJI and determined factors associated with mycobacterial PJI. No statistically significant differences of baseline characteristics and co-morbidities were detected (Table 1). The duration and onset of RGM infection after prosthesis implantation was significantly different from that of culture-proven non-mycobacterial infections (*p* = .001 and < .001, respectively). Subjects with RGM PJI had a significantly shorter infection duration and a significantly shorter time from implantation to infection onset while subjects with non-mycobacterial PJI had a delayed to late onset of infection after arthroplasty. Clinical presentations, peripheral and synovial WBC counts, ESR and CRP levels were not significantly different. A significantly larger proportion of patients with RGM PJI received an inappropriate antibiotic regimen as their initial treatment (*p* = .005). All subjects with RGM PJI received combination oral antibiotics as maintenance therapy (*p* < .001). The duration of maintenance therapy was significantly longer in patients with RGM PJI (365 vs. 134 days, *p* = .025). Overall surgical interventions were performed on 174 subjects (97.8%). Surgical prosthesis removal accounted for the majority of interventions, with two-stage surgery performed in 52.4% of cases and resection arthroplasty performed in 27.5% of cases. Joint arthrodesis and one-stage surgery were performed in 1.1% and 0.6% of cases, respectively. A total of 150 (84.3%) and 130 (73%) subjects complied with clinical follow-up 6 months and 12 months after infection, respectively. Favorable outcomes at 6 and 12 months were comparable between groups.

**Table 1:** Demographics, clinical characteristics and outcomes between culture-proven non-mycobacterial and rapidly growing mycobacterial (RGM) prosthetic joint infection

	Culture-proven non-mycobacterial PJI (*n* = 111)	RGM PJI (n = 11)	*P* value
Age, mean ± SD (yrs)	63.5 ± 13.7	66.27 ± 9.5	.505
Gender, no. (%)			
Male	40 (36)	3 (27.3)	.745
Female	71 (64)	8 (72.7)	
BW (kg), mean ± SD	64.3 ± 11.7	58.3 ± 9.9	.104
BMI (kg/m^2^), mean ± SD	25.6 ± 4.2	25.2 ± 4.0	.798
BMI ≥25, no. (%)	27/57 (47.4)	5/8 (62.5)	.475
Site of PJI, no. (%)			
Knee	67 (60.4)	10 (90.9)	.053
Hip	44 (39.6)	1 (9.1)	
Prior joint disease, no. (%)			
Primary degenerative	64 (57.7)	9 (81.8)	.197
Secondary degenerative^a^	47 (42.3)	2 (18.2)	
Comorbidity, no. (%)			
Diabetes mellitus	25 (22.5)	2 (18.2)	1.0
Chronic kidney disease	12 (10.8)	1 (9.1)	1.0
Chronic lung disease	2 (1.8)	0(0)	1.0
Chronic liver disease	6 (5.4)	0 (0)	1.0
Cardiovascular disease	54 (48.6)	4 (36.4)	.436
Malignancy	4 (3.6)	0 (0)	1.0
Immnunosuppressive	3 (2.7)	0 (0)	1.0
No immnunosuppressive	108 (97.3)	11 (100)	
Rheumatologic disease	4 (3.6)	0 (0)	1.0
Duration, median (min, max) (days)^b^	330 (4, 10,220)	60 (19, 2675)	.001
Onset, no. (%)			
≤ 90 days	19 (17.1)	9 (81.8)	< .001
> 90 days	92 (82.9)	2 (18.2)	
Clinical presentations, no. (%)	110 (99.1)	11 (100)	1.0
Pain	99 (89.2)	10 (90.9)	1.0
Loosening	26 (23.4)	0 (0)	.118
Sinus drainage	41 (36.9)	6 (54.5)	.333
Fever	34 (30.6)	2 (18.2)	.504
Radiological loosening	36/55 (65.5)	1/6 (16.7)	.031
Peripheral WBC count, median (min, max) (cell/mm^3^) Log WBC count, mean ± SD	8270 (3020, 42,500) 9.1 ± 0.4	7220 (6320, 14,430) 9.0 ± 0.2	.066 .215
Cr, median (min, max) (mg/L)	0.8 (0.2, 4.4)	0.8 (0.3, 1.6)	.634
ESR, mean ± SD (mm/h)	89.6 ± 29.9	92.6 ± 29.7	.758
CRP, median (min, max) (mg/L)	46.3 (3, 401)	50.9 (3, 117)	.733
Synovial fluid WBC, median (min, max) (cell/mm^3^)	37,700 (3050, 300,800)	1100 (1100, 1100)	.109
Periprosthetic tissue cultures, no. (%)			
Single isolate growth	87 (80.6)	11 (100)	.560
≥ 2 isolate growth	12 (11.1)	0 (0)	
No growth	9 (8.3)	0 (0)	
Initial ATB therapy, no. (%)			
Appropriate	52 (47.7)	0 (0)	.005
Inappropriate	57 (52.3)	11 (100)	
Duration, median (min, max) (days)	47 (6, 270)	42 (14, 129)	.272
Maintenance oral ATB therapy, no. (%)			
Single	62 (75.6)	0 (0)	< 0.001
Combined	20 (24.4)	11 (100)	
Duration, median (min, max) (days)	134 (14, 605)	365 (60, 910)	.025
Surgical treatment, no. (%)			
Retention of prosthesis	19 (17.8)	1 (9.1)	.688
Removal of prosthesis	88 (82.2)	10 (90.9)	
Outcomes at 6 months, no. (%)			
Favorable	69 (79.3)	6 (66.7)	.405
Unfavorable	18 (20.7)	3 (33.3)	
Outcomes at 12 months, no. (%)			
Favorable	58 (77.3)	5 (83.3)	1.0
Unfavorable	17 (22.7)	1 (16.7)	

*Abbreviations*: *ATB* antibiotics, *BMI* body mass index, *BW* body weight, *Cr* creatinine, *CRP* Creactive protein, *ESR* erythrocyte sedimentation rate, *PJI* prosthetic joint infection, *SD* standard deviation, *WBC* white blood cell

^a^Including prior surgery, previous fracture, avascular necrosis, rheumatoid arthritis, post infection, gout and tumor

^b^Temporal duration from prosthesis implantation to clinical diagnosis of PJI

### Causative agents of prosthetic joint infections

Rapidly growing mycobacteria (*M. fortuitum*, *M. abscessus*, *M. peregrinum*) and *M. tuberculosis* were responsible for the mycobacterial PJIs (Table 2). Two subjects had a co-infection: one with a coagulase-negative staphylococcus and the other with methicillin-susceptible *Staphylococcus aureus*. Staphylococci (34.3%) were the most common causes of culture-proven non-mycobacterial PJIs, and gram-negative bacilli were detected in 17.4% of cases. Twelve subjects from the culture-proven non-mycobacterial PJI group had 2 or more microorganisms detected in their cultures. No organisms were identified in 51 cases (28.7%).

**Table 2:** Organisms identified in the prosthetic joint infections included in this study^a,b^

Etiologic organism	Organism	n (%)
Mycobacteria	*Mycobacterium tuberculosis* *Mycobacterium fortuitum* *Mycobacterium abscessus* *Mycobacterium peregrinum*	5 (2.8) 9 (5.0) 1 (0.6) 1 (0.6)
Bacteria	*Staphylococcus aureus* (methicillin susceptible) Coagulase negative staphylococci *Staphylococcus aureus* (methicillin resistant) Enterococci *Pseudomonas aeruginosa* *Klebsiella pneumoniae* *Streptococcus pyogenes* *Salmonella* spp. *Escherichia coli* *Streptococcus agalactiae* *Enterobacter* spp. Group D streptococci *Peptostreptococcus* spp. *Proteus* spp. Other gram-negative rods Other streptococci	30 (16.9) 16 (9.0) 15 (8.4) 13 (7.3) 10 (5.6) 6 (3.4) 4 (2.2) 4 (2.2) 4 (2.2) 4 (2.2) 3 (1.7) 2 (1.1) 1 (0.6) 1 (0.6) 3 (1.7) 5 (2.8)
Fungi	*Candida* spp.	3 (1.7)
Culture-negative		51 (28.7)

^a^Cultures included synovial fluid aspirates and periprosthetic tissue samples

^b^Fourteen subjects were culture-positive for ≥2 microorganisms

### Factors associated with rapidly growing mycobacterial (RGM) prosthetic joint infection

The onset of PJI, especially PJI that developed within 90 days of arthroplasty (early-onset PJI), was significantly associated with RGM infection (OR 21.86; 95% CI 4.25–112.30; *p* < .001). Body weight and log-transformed peripheral WBC counts were not associated with mycobacterial PJI in the multivariate analysis (Table 3).

**Table 3:** Factors associated with rapidly growing mycobacterial prosthetic joint infection^a,b^

Factors	Univariate OR (95% CI)	*P* value	Multivariate OR (95% CI)	*P* value
Body weight	0.95 (0.90–1.01)	.109	–	–
Onset				
≤90 days	21.79 (4.36–109.0)	< .001	21.86 (4.25–112.30)	< .001
>90 days	1		1	
Log WBC count ^*c*^	0.33 (0.06–1.89)	.211	–	–

*Abbreviations*: *CI* confidence interval, *OR* odds ratio, *WBC* white blood cell

^a^Data obtained from subjects with culture-proven non-mycobacterial and rapidly growing mycobacterial PJI

^b^Analyzed by univariate and multivariate logistic regression (forward stepwise)

^c^Peripheral white blood cell count

### Factors associated with a favorable outcome

All subjects who complied with 6-month and 12-month follow-ups were analyzed to identify factors associated with a favorable outcome. Our 6-month outcome analysis included 150 patients. Of these patients, 79 were in remission, 37 were stable, 16 had relapsed, 3 had a re-infection and 15 failed treatments. Prosthesis removal was more commonly associated with a favorable outcome 6 months after diagnosis (OR 5.96; 95% CI 1.88–18.88; *p* < .01) (Table 4). Our 12-month outcome analysis included 130 patients. Of these, 77 were in remission, 28 were stable, 6 had relapsed, 2 had re-infections, and treatments failed in 17. Prosthesis removal was again significantly associated with a favorable outcome 12 months after infection (OR 3.96; 95% CI 1.15–13.71; *p* = .03). Body mass index (BMI) was associated with poor outcomes at both the 6- and 12-month time points (Table 4). Pathogen type was not found to be associated with a favorable outcome (data not shown).

**Table 4:** Factors associated with favorable outcomes^a,b^

Factors	Univariate OR (95% CI)	*P* value	Multivariate OR (95%CI)	*P* value
At 6 months				
Body mass index	0.88 (0.78–0.98)	.02	0.86 (0.77–0.97)	.02
Creatinine	0.64 (0.33–1.28)	.21	–	–
Chronic kidney disease	0.43 (0.13–1.41)	.16	–	–
Malignancy	0.18 (0.29–1.13)	.07	–	–
Combined maintenance antibiotic therapy	0.38 (0.15–0.93)	.04	–	–
Removal of prosthesis	2.76 (1.10–6.88)	0.03	5.96 (1.88–18.88)	< .01
At 12 months				
Gender (Female)	1.82 (0.76–4.34)	.18	–	–
Age	0.97 (0.93–1.01)	.13	–	–
Body mass index	0.89 (0.77–0.99)	.05	0.87 (0.76–0.99)	.04
Creatinine	0.48 (0.20–1.15)	.10	–	–
Combined maintenance antibiotic therapy	3.41 (0.75–15.50)	0.11	–	–
Removal of prosthesis	4.33 (0.58–32.33)	0.15	3.96 (1.15–13.71)	.03

*Abbreviations*: *CI* confidence interval, *OR* odds ratio

^a^Obtained from subjects with culture-proven non-mycobacterial, mycobacterial and culture-negative PJI

^b^Analyzed by univariate and multivariate logistic regression (forward stepwise)

### Clinical features of mycobacterial prosthetic joint infection from 16 subjects

Of 178 total subjects, only 16 (9%) were confirmed by culture to have a mycobacterial infection. The clinical characteristics, *Mycobacterium* species, treatment modalities and 12-month outcomes of those subjects who were diagnosed with mycobacterial PJI are shown in Additional file 1: Table S1. For RGM isolates, antimicrobial susceptibility tests were performed in 8 out of 11 patients. The isolates were susceptible to amikacin 100%, ciprofloxacin 100%, clarithromycin 50%, doxycycline 14%, imipenem 13%, and co-trimoxazole 13%. None of the isolates were susceptible to cefoxitin.

## Discussion

Of 178 subjects, 16 (9%) had PJI caused by mycobacterial infection. Mycobacterial PJI in this study was classified into 2 categories: 1) PJI caused by RGM namely *M. fortuitum*, *M. abscessus* and *M. peregrinum*; 2) PJI caused by MTB. The pathogenesis of the two types of mycobacterial PJI is completely different. RGM PJI are likely associated with intra-operative contamination [10] or disseminated infection in immunocompromised patients [11], whereas a local reactivation of dormant foci or hematogenous spreading is a feasible etiology for MTB PJI. RGM were the leading etiologic agents of mycobacterial PJI. Thus, this study focuses on the clinical characteristics and factor associated with RGM PJI. Of the 16 subjects diagnosed with mycobacterial PJI, 7 subjects had at least 1 of the following conditions: systemic lupus erythematosus (SLE), chronic corticosteroid therapy, chronic liver disease, diabetes mellitus, leukemia or chronic kidney disease. These conditions may be predisposing factors for mycobacterial infection [12–14]. However, the present study found no comorbidities that were significantly associated with mycobacterial PJI similar to several previously published studies [10, 15–17].

Early-onset PJI was significantly associated with RGM PJI in this study. Most of RGM PJI cases had an early-onset infection, which was markedly faster than the onsets reported in a previous study. Eid et al. [8] found the median onset of RGM PJI to be 312 weeks after prosthesis implantation. The shorter onset of RGM PJI in our study may relate to intra-operative contamination. However, this retrospective study is not able to find the source of RGM in our patients. A previous study discovered a cluster of genetically identical early-onset *M. fortuitum* PJIs, but a common source of these infections was not identified [10]. An association between an early-onset RGM PJI and intra-operative contamination therefore remains controversial. Five subjects with MTB PJI showed delayed to late onset of infection similar to a previous systematic review [14]. Distant or local foci of active tuberculosis were not recognized in the MTB subjects.

Periprosthetic tissue and synovial fluid cultures as well as histopathology are warranted in the diagnosis of mycobacterial PJI. All cases of mycobacterial PJI in this study were diagnosed using periprosthetic tissue cultures, including 3 subjects with mycobacterial culture-positive synovial fluid. Previous case reports have noted that periprosthetic tissue cultures have a high sensitivity in the diagnosis of mycobacterial PJI [16–20]. In the isolation of mycobacteria from periprosthetic tissue, several critical points should be considered. RGM were able to grow on routine bacterial culture media such as blood agar and MacConkey agar [10, 15, 17, 18]. However, RGM culture growth may be delayed at least 5–7 days, a time frame in which routine specimens may have already been discarded. Moreover, RGM manifests as weakly gram-positive bacilli on culture and has occasionally been found to resemble corynebacteria [21]. In contrast, MTB is hard to isolate on routine bacterial cultures. Special solid or liquid media for mycobacteria and a prolonged incubation time are required to isolate mycobacteria [21]. As such, when mycobacterial PJI is suspected, such as those PJIs with negative cultures, periprosthetic tissue samples should be submitted for mycobacterial culture in addition to another attempted bacterial isolation.

The mycobacterial PJI group received inappropriate initial antibiotics significantly more often than patients with non-mycobacterial PJI. A low index of suspicion and negative routine cultures could be the reason patients in the mycobacterial PJI group received inappropriate initial antibiotics. Antibiotic susceptibility testing (AST), combination antimicrobial therapy and a prolonged course of treatment are indicated in the management of mycobacterial PJI. RGM are usually resistant to traditional first-line anti-tuberculosis drugs. Yang et al. [22] showed that almost all *M. fortuitum* group, *M. chelonae* and *M. abscessus* were susceptible to amikacin; > 60%–80% of isolates were susceptible to clarithromycin, fluoroquinolones, and imipenem. The efficacies of cefoxitin, doxycycline and tobramycin against the 3 RGM were unsatisfactory [22]. However, there is no standardized treatment protocol or study correlating in vitro pathogen susceptibility and the clinical response of specific antibiotics directed towards RGM [11]. The majority of our patients with RGM PJI exhibited a satisfactory response with empirical treatment using amikacin in combination with cefoxitin, followed by combined fluoroquinolone and clarithromycin regimens. In this study, the treatment regimens in 5 subjects with MTB PJI were individualized because of drug intolerance, drug hypersensitivity and resistant organisms complicated treatment. This study revealed that subjects with mycobacterial PJI received a long duration of maintenance antimicrobial therapy, which is necessary to prevent recurrent infection.

Debridement and implant removal are required in the treatment of PJI. Removal of the infected prosthesis was a factor associated with a significantly increased favorable outcome rate 6 and 12 months after infection diagnosis in this study. Ninety-one subjects (52.4%) underwent two-stage surgery, which involves debridement and removal of all infected prosthetic components, insertion of antibiotic cement spacer, followed by timely re-implantation of a new prosthesis [9]. Two-stage surgery was associated with successful treatment of both bacterial [23] and fungal [24] PJI. The role of antibiotic cement spacer in this setting is to deliver high concentration of antibiotics directly to the infected site and to maintain limb length by preventing soft tissue contracture [25]. However, the optimal surgical strategy for treating mycobacterial PJI is still unknown. A case series evaluating *M. fortuitum* PJI found that the removal of the infected prosthesis combined with a prolonged course of antibiotics generally led to good outcomes [26]. A previous study evaluating MTB PJI of the knee concluded that late onset of infection (> 6–8 weeks), presence of a draining sinus, concomitant bacterial infection, osteolysis or prosthesis instability indicated the need to remove the infected prosthesis [27]. Obesity may diminish treatment success, as BMI was a factor significantly associated with an unfavorable outcome in this study.

The present study had some limitations. The retrospective design prevented us from assessing several variables, such as HIV status, a history of previous TB, previous antibiotic treatment from outside hospitals and overall therapeutic compliance. The prevalence of mycobacterial PJI and culture-proven non-mycobacterial PJI might be underestimated owing to the culture-negative PJI group. A number of subjects with culture-negative PJI received empirical antimicrobial therapy prior to obtaining their synovial tissues for culture, which hampered the sensitivity of pathogen identification. Patients with PJI, the definite favorable outcome can be judged after a follow-up of at least 2 years. The favorable outcome was followed up only within 12 months in this study. The 6-month and 12-month outcomes were not completely evaluated in 6 of 16 mycobacterial PJIs owing to a transfer out of our hospital or loss to follow-up. Future long-term prospective cohort studies should be conducted to more accurately identify factors associated with successful and unsuccessful treatment outcomes.

## Conclusions

Mycobacteria, particularly RGM are important pathogens causing PJI. The clinical characteristics of mycobacterial PJI are indistinguishable from those of culture-proven non-mycobacterial PJI. RGM usually cause an early onset infection. Additional cultures for mycobacteria are an optimal front-line diagnostic protocol for those with suspected PJI with an early onset of infection, a negative routine culture or refractory to antibiotics. Removal of the infected implant significantly improves the chance of a favorable outcome.

## Additional file

Additional file 1: Table S1. (Clinical features and outcomes of 11 non-tuberculous mycobacterial and 5 tuberculous prosthetic joint infections). (DOC 67 kb)

## Abbreviations

AST: Antibiotic susceptibility testing
BMI: Body mass index
CRP: C-reactive protein
ESR: Erythrocyte sedimentation rate
HIV: Human immunodeficiency virus
IDSA: The Infectious Diseases Society of America
MGIT: Mycobacterial growth indicator tube
MTB: *Mycobacterium tuberculosis*
PJI: Prosthetic joint infection
RGM: Rapidly growing mycobacteria
SLE: Systemic lupus erythematosus
TB: Tuberculosis
THA: Total hip arthroplasty
TKA: Total knee arthroplasty
WBC: White blood cell
