# Supplementary material for: Rapidly-growing mycobacterial infection: a recognized cause of early-onset prosthetic joint infection
Source: BMC Infect Dis. 2017 Dec 28;17:802. doi: 10.1186/s12879-017-2926-3 (PMC5745588; doi:10.1186/s12879-017-2926-3)
Supplement: Additional file 1: Table S1. — (Clinical features and outcomes of 11 non-tuberculous mycobacterial and 5 tuberculous prosthetic joint infections). (DOC 67 kb) [file 12879_2017_2926_MOESM1_ESM.doc]

**Additional file**

**Table S1.** Clinical features and outcomes of 11 non-tuberculous mycobacterial and 5 tuberculous prosthetic joint infections

| Case  No. | Age (yrs.)  /Sex | Underlying  diseases | Site | Duration  (days)^a^ | Symptoms | | Peripheral  WBC^b^  (cells/mm^3^) | ESR (mm/hr)/  CRP (mg/L)^b^ | Causative pathogens | Specific ATB  regimens^c^ | Type of  surgery | Outcome^d^ |
| --- | --- | --- | --- | --- | --- | --- | --- | --- | --- | --- | --- | --- |
| 1 | 68/F | Gout | Knee | 60 | Pain, fever | | 7,290 | 111/117 | *Mycobacterium*  *fortuitum* | IV FOX, AMK then oral CIP, SMX/TMP and DOX | Two-stage  exchange | Remission |
| 2 | 64/M | DM, HT | Hip | 2,675 | Sinus drainage | | 14,430 | 98/64.1 | *Mycobacterium*  *fortuitum* | IV FOX, AMK then oral CLA, CIP and RIF | Two-stage  exchange | Remission |
| 3 | 58/F |  | Knee | 30 | Pain, fever | | 7,220 | 140/87.7 | *Mycobacterium*  *fortuitum* | IV FOX and AMK then oral CLA, CIP and ETB | Two-stage  exchange | NA |
| 4 | 77/F | HT | Knee | 60 | | Pain, sinus  drainage | 10,240 | 111/35.8 | *Mycobacterium*  *fortuitum* | IV FOX, AMK then oral CLA, CIP | Two-stage  exchange | Remission |
| 5 | 56/F |  | Knee | 30 | Pain | | 6,320 | 72/50.9 | *Mycobacterium*  *fortuitum* | IV AMK then oral CLA, CIP | Two-stage  exchange | Remission |
| 6 | 69/F | DM, CKD | Knee | 180 | Pain | | 6,530 | 65/3 | *Mycobacterium*  *fortuitum* | IV LVX, AMK then oral CLA, CIP | Two-stage  exchange | Failure |
| 7 | 80/F | DM, HT | Knee | 19 | Pain, sinus drainage | | 6,820 | 73/37.8 | *Mycobacterium*  *fortuitum* | IV IMI, CIP, AMK then oral CIP, DOX | Debridement,  retention of  prosthesis | NA |

**Table S1** (continued)

| Case  No. | Age (yrs.)  /Sex | Underlying  diseases | Site | Duration  (days)^a^ | | Symptoms | Peripheral  WBC^b^  (cells/mm^3^) | ESR (mm/hr)/  CRP (mg/L)^b^ | Causative pathogens | Specific ATB  regimens^c^ | Type of surgery | Outcome^d^ |
| --- | --- | --- | --- | --- | --- | --- | --- | --- | --- | --- | --- | --- |
| 8 | 51/M | Gout | Knee | 90 | | Pain, sinus drainage | 6,720 | 34/23.8 | *Mycobacterium*  *fortuitum* | IV AMK then oral CLA, CIP | Resection  arthroplasty | NA |
| 9 | 76/F | HT | Knee | 90 | | Pain | 7,000 | 105/66 | *Mycobacterium*  *fortuitum* | IV FOX, CIP then oral CLA, CIP | Resection arthroplasty | Remission |
| 10 | 59/M |  | Knee | 19 | Pain, sinus  drainage | | 7,500 | 119/66 | *Mycobacterium*  *abscessus* | IV FOX then oral  CLA and CIP | Two-stage  exchange | NA |
| 11 | 71/F |  | Knee | 30 | Pain, sinus drainage | | 7,500 | 90/24.19 | *Mycobacterium*  *peregrinum* | IV FOX, AMK then oral CLA, SMX/TMP | Resection  arthroplasty | NA |
| 12 | 54/F | SLE, CLD, on steroid | Hip | 3,285 | | Pain, loosening | 4,880 | 45/5.86 | *Mycobacterium*  *tuberculosis* | INH, ETB | Resection arthroplasty | Remission |
| 13 | 79/F | DM, HT | Knee | 940 | | Pain | 11,200 | 94/63 | *Mycobacterium*  *tuberculosis* | INH, ETB and PZA | Debridement,  retention of  prosthesis | Relapse |
| 14 | 49/M | Leukemia | Hip | 3,650 | | Pain, loosening | 6,250 | 115/110 | *Mycobacterium*  *tuberculosis, CoNS* | IV CIP then oral RIF, OFX and PZA | Two-stage  exchange | NA |

**Table S1** (continued)

| Case  No. | Age (yrs.)  /Sex | Underlying  diseases | Site | Duration  (days)^a^ | Symptoms | Peripheral  WBC^b^  (cells/mm^3^) | ESR (mm/hr)/  CRP (mg/L)^b^ | Causative pathogens | Specific ATB  regimens^c^ | Type of surgery | Outcome^d^ |
| --- | --- | --- | --- | --- | --- | --- | --- | --- | --- | --- | --- |
| 15 | 83/M | CKD, HT | Knee | 300 | Pain | 4,360 | 23/10.4 | *Mycobacterium*  *tuberculosis* | RIF, INH, ETB | Debridement,  retention of  prosthesis | Remission |
| 16 | 61/M |  | Hip | 210 | Pain, sinus drainage | 9,410 | 54/123 | *Mycobacterium*  *tuberculosis*, MSSA | RIF, INH, ETB | Resection arthroplasty | Remission |

^a^ Temporal duration from prosthesis implantation to clinical diagnosis of PJI

^b^ Peripheral white blood cell count, ESR, and CRP at initial presentation

^c^ Specific antibiotics that were prescribed after establishing a definite diagnosis

^d^ Clinical outcomes of follow-up through 12 months

Abbreviations: AMK, amikacin; ATB, antibiotics; CIP, ciprofloxacin; CKD, chronic kidney disease; CLA, clarithromycin; CLD, chronic liver disease; CoNS, coagulase negative staphylococcus; CRP, C-reactive protein; DM, diabetes mellitus; DOX, doxycycline; ESR, erythrocyte sedimentation rate; ETB, ethambutol; F, female; FOX, cefoxitin; HT, hypertension; IMI, imipenem; INH, isoniazid; IV, intravenous; LVX, levofloxacin; M, male; MSSA, methicillin sensitive *Staphylococcus aureus*; NA, not available; OFX, ofloxacin; PZA, pyrazinamide; RIF, rifampicin; WBC, white blood cell; SLE, systemic lupus erythematosus; SMX/TMP, sulfamethoxazole/trimethoprim
